# Investigating young children’s physical activity through time and place

**DOI:** 10.1186/s12942-024-00373-8

**Published:** 2024-05-14

**Authors:** T. Remmers, P. Koolwijk, I. Fassaert, J. Nolles, W. de Groot, S. B. Vos, S. I. de Vries, R. Mombarg, D. H. H. Van Kann

**Affiliations:** 1 School of Sport Studies, Fontys University of Applied Sciences, Eindhoven, the Netherlands; 2 Research group Healthy Lifestyle in a Supporting Environment, The Hague University of Applied Sciences, The Hague, The Netherlands; 3 Institute of Sport Studies, Hanze University of Applied Sciences, Groningen, the Netherlands; 4 Department of Industrial Design, Eindhoven University of Technology, Eindhoven, the Netherlands; 5 Department of Public Health and Primary Care, Leiden University Medical Centre, Health Campus The Hague, The Hague, The Netherlands; 6 Faculty of Orthopedagogy, University of Groningen, Groningen, the Netherlands; 7 Department of Health Promotion, Maastricht University, Maastricht, the Netherlands; 8 Faculty of Behavioural and Movement Sciences, Vrije Universiteit Amsterdam, Amsterdam, The Netherlands

**Keywords:** Accelerometer, GPS, GIS, Primary school, Transport

## Abstract

**Background:**

Previous research indicates the start of primary school (4-5-year-old) as an essential period for the development of children’s physical activity (PA) patterns, as from this point, the age-related decline of PA is most often observed. During this period, young children are exposed to a wider variety of environmental- and social contexts and therefore their PA is influenced by more diverse factors. However, in order to understand children’s daily PA patterns and identify relevant opportunities for PA promotion, it is important to further unravel in which (social) contexts throughout the day, PA of young children takes place.

**Methods:**

We included a cross-national sample of 21 primary schools from the Startvaardig study. In total, 248 children provided valid accelerometer and global positioning (GPS) data. Geospatial analyses were conducted to quantify PA in (social) environments based on their school and home. Transport-related PA was evaluated using GPS speed-algorithms. PA was analysed at different environments, time-periods and for week- and weekend days separately.

**Results:**

Children accumulated an average of 60 min of moderate-to-vigorous PA (MVPA), both during week- and weekend days. Schools contributed to approximately half of daily MVPA during weekdays. During weekends, environments within 100 m from home were important, as well as locations outside the home-school neighbourhood. Pedestrian trips contributed to almost half of the daily MVPA.

**Conclusions:**

We identified several social contexts relevant for children’s daily MVPA. Schools have the potential to significantly contribute to young children’s PA patterns and are therefore encouraged to systematically evaluate and implement parts of the school-system that stimulate PA and potentially also learning processes. Pedestrian trips also have substantial contribution to daily MVPA of young children, which highlights the importance of daily active transport in school- and parental routines.

## Background

Early childhood (i.e. from birth until five years old) has recently become a prominent health-promotion target group as there is increased recognition that establishing health-supporting environments in early childhood can reduce subsequent population-level risk factors and disease [1]. Within these health-supporting environments, physical activity (PA) and sedentary behaviours contribute to the development of children’s physical-, psychosocial and cognitive abilities [2–4]. The consistency, quality and timing of these interconnected behaviours are formed in early childhood and the accompanied habits tend to track from childhood through adolescence [5, 6].

In early childhood, the role of PA is of particular interest because through PA a child interacts with the surrounding environment and experiences the capabilities of its own body. By doing so, PA acts as an initiator of various learning processes [7]. In addition, sufficient- and appropriate variation of PA leads to the development of fundamental motor skills [8] which are important building blocks for more complex motor skills later in life [9–11]. Research suggests that PA and the development of motor skills may be more intertwined with cognitive development than previously assumed [12–14]. In addition, more PA in early childhood is associated with a broad range of favourable indicators relating to cardiometabolic-, skeletal- and morphological health [15–17]. In 2020, the WHO formulated specific international guidelines for early childhood [18]. For 3-4-year-old children, at least 180 daily minutes of PA (of which 60 min of moderate-to-vigorous intensity) and no more than 60 min of daily sedentary screen time are recommended [19]. Before five- to six years of age, children seem to be sufficiently active, especially at light intensity [20, 21]. However, already around the age of 6 years, children’s PA levels decline while sedentary activities such as screen-related behaviours increase [21]. To understand the mechanism behind this age-related decline, it is vital to gain more insight in the daily PA patterns of young children [22–24].

Previous longitudinal studies showed that the onset of primary school is crucial in the development of healthy PA patterns of children, as notable increases in sedentary patterns were observed in this phase [25, 26]. In primary school, children are exposed to a wider social- and physical environment (both in- and out of school), extending the potential of barriers and affordances for PA. Also, previous research showed that sedentary time predominantly increased during school hours, suggesting that in-school practices are probably responsible for decreasing PA [27–29]. Other studies have reported that variability between children’s PA was highest out of school [28, 30, 31]. This illustrates that the start of primary school is an interesting phase in which a complex and dynamic system of environmental factors have great influence of children’s emerging PA patterns [26, 32, 33]. In addition, the context in which PA occurs greatly influences the potential of these factors in influencing PA [34]. For example, children’s PA at school and PA at home are influenced by different environmental factors [34]. This means that in order to understand children’s PA patterns and how to effectively promote PA, more contextual information about the type of PA is essential [35]. However, investigating context-specific PA of young children is complex, because they predominantly perform PA in short sporadic bursts, sometimes without clear motives [36]. This makes the application of subjective assessment (e.g. parental recall) challenging and susceptible for social-desirability bias [37, 38]. On the other hand, objective measurements (e.g. accelerometry) fail to capture essential contextual information (e.g. location) about the type of PA performed [16]. One way of overcoming these issues is by combined accelerometer and GPS methodology, which simultaneously combines information about PA and the geographic context [39]. Previous studies that have used this methodology in young children are scarce and have either focused on places for PA within childcare centers [40] or residential neighbourhoods separately [41]. Results showed that within childcare centers, larger open areas with portable equipment (e.g. balls, toys) were associated with children’s PA-hotspots [40] and that approximately 60% of the daily moderate-to-vigorous PA (MVPA) of 3-year-old Western-Australian children occurred < 500 m from their home, while 30% of daily MVPA occurred outside their neighbourhood (> 1600 m from their home) [41]. Although this provides valuable insights in where children’s PA takes place within the childcare and neighbourhood context, integrated information from both contexts is warranted to evaluate the degree to which each of these contexts contribute to children’s daily PA. Therefore, the purpose of this study was to investigate context-specific PA patterns of 4-6-year-old children (i.e. onset of primary school in the Netherlands) to improve our understanding of how to effectively promote these PA patterns.

## Methods

### Design and participants

**Fig. 1:**
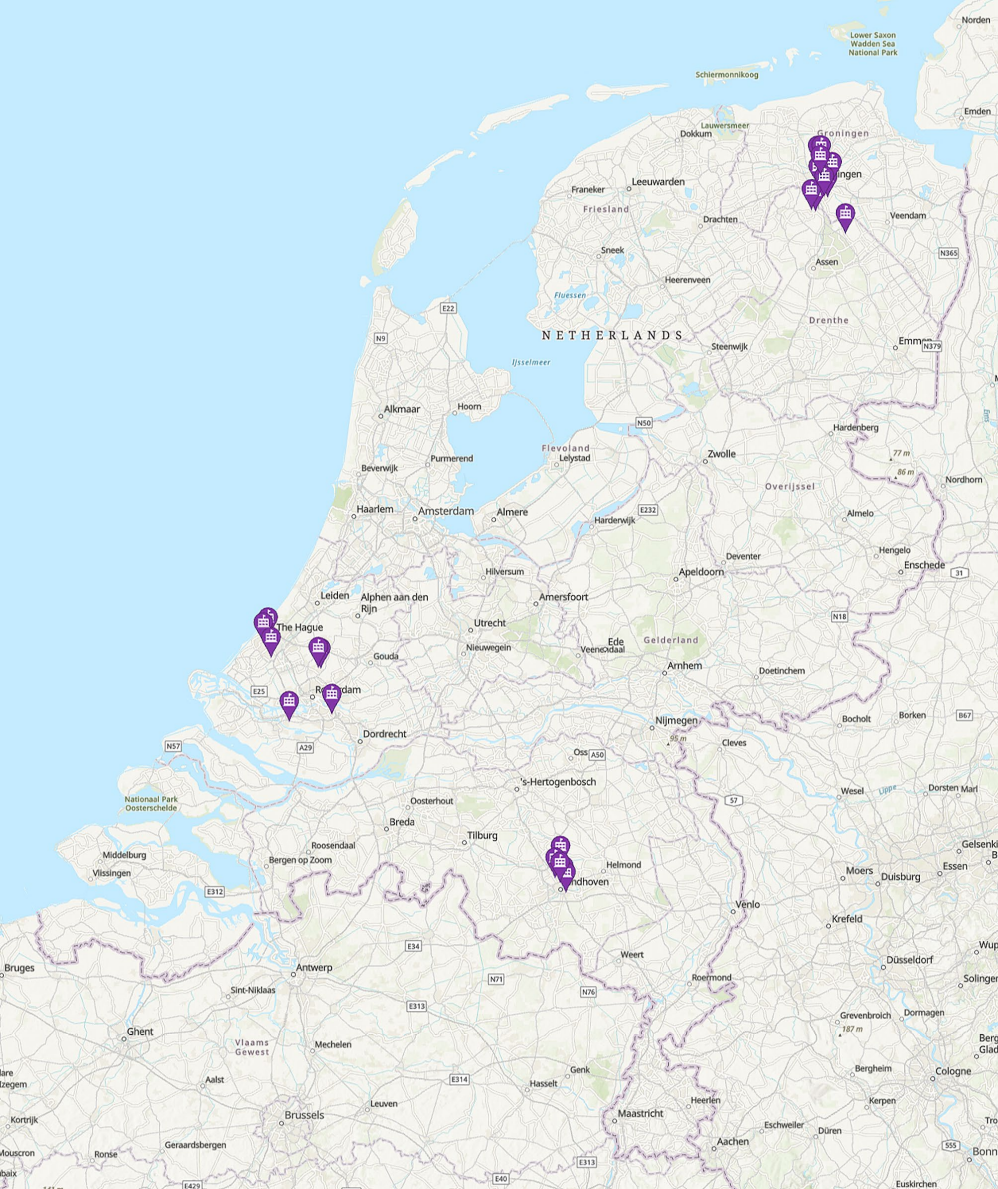
Geographical distribution of participating schools

In this cross-sectional study, a convenience sample of 21 primary schools in medium- to large scale cities of the Netherlands (i.e. 5 schools located in Eindhoven, 7 schools in the vicinity of The Hague, 9 schools in the vicinity of Groningen) were selected from the cross-national ‘Start Vaardig’ project (Dutch for ‘Skilful Start’). The three cities lie relatively close to each other (i.e. 370 km of driving distance to visit all three cities), with comparable climate during the period of measurement. Participating schools represented a wide variation of predominantly suburban areas in the north, middle, and south of the Netherlands (Fig. 1). In terms of PA- or transport related geography (e.g. percentage greenness, flat land, degree of urbanization) the suburban areas of the participating schools were comparable. The Dutch primary school system ranges from grade 1 (for 4-year-old children) till grade 8 (for 12-year-old children), and in our study children from grades 1 and 2 were eligible for participation. Schools provided detailed information about schedules and break times.

All participating schools were visited four times by a team of two trained researchers. At the first visit, children and teachers were informed about the project and shown how to wear the accelerometer (Actigraph GT3X+, Pensacola, FL, USA) and GPS devices (Qstarz BT1000XT, Tapei, Taiwan). Children were provided with a written information letter and informed consent form. Parents were given the possibility to sign and return the written informed consent form to their child’s teacher or to sign online. Teachers were provided with additional written instruction about the purpose of the project and how to collect the informed consent forms. At the second visit, consent forms were collected, and reminders were handed out to the children. At the third visit, children received the devices with verbal instruction and parents were provided with written instructions. We instructed children to wear the devices at the right hip using an elastic belt during waking hours, for six consecutive days (containing two weekend days). We instructed to only remove the devices during sleep or water-related activities (e.g. swimming, showering) and to recharge the GPS logger every day just before going to bed. Additionally, parents of all participating children received a paper questionnaire, as well as an online version of the questionnaire. At the fourth and last visit, devices were returned, and paper questionnaires were collected.

Data collection took place between the 26th of May and the 15th of July 2021, in-between restrictions caused by the COVID-19 pandemic. Daily average temperature was 18.1 degrees Celsius (SD = 3.1) with average precipitation of 3.7 mm per day (69% of days with < 1 mm). Sunset times during this period were between 21:38 and 21:48 h (www.timeanddate.com). Ethical approval was obtained by the Ethical Research Committee of the VU Medical Centre in Amsterdam, the Netherlands (VCWE-2020-137).

### Measurements

Parents provided socio-demographic information in the questionnaire, such as their child’s date of birth and gender, postal code and number of spouses. In addition, questions were asked about the frequency and reason that the accelerometer- and GPS devices were taken off (e.g. swimming, showering, discomfort) as well as the days on which their child did not sleep at home during the night.

Numerous studies have supported validity and accuracy of the accelerometer and GPS devices [42–45]. We used the manufacturer’s software to initialize devices and export data to CSV-files, for the accelerometer (Actilife version 6.13.4) and GPS logger (QTravel version 1.54) separately. Devices were set to record data every 10 s epochs. GPS loggers were initialized to record data between 6 AM and 10 PM to optimize battery life and storage capacity and to stop logging when storage capacity was full. We processed accelerometer data using R-package GGIR (version 3.0–1) [46], which included algorithms regarding autocalibration of accelerometers [47] and standard weartime detection algorithms. Namely, non-wear time was investigated per 15-minute time blocks, while the definition of non-wear time was based on the standard deviation (i.e. <13 milli gravity (mg)) and range (i.e. <50 mg) of the 60-minute time window that centered each 15-minute time block. Intensity-classification of PA was based on the vertical-axis classification of Evenson et al. (2008) [48] and were adjusted for the 10 s epoch by linear interpolation. We processed combined GPS and accelerometer output using the HABITUS (hbGPS) software [49, 50], inspired by functionality from the earlier PALMS system [44, 51]. GPS data was cleaned by removing outliers based on (1) missing values in speed estimates, (2) speed greater than 130 km/h with a speed-difference > 30 km/h, and (3) elevation change between successive values > 1000 m [50]. Trips were identified by a consistent speed of at least 1 kmph for any sequence of three successive datapoints (i.e. 30 s). We furthermore identified trip pause points with insufficient speed (see sentence above) for a maximum of 2 min. When the pause time exceeded 2 min, we classified this as a trip end point. Alternatively, we treated this as one common trip. We subsequently removed trips with (1) distance < 100 m, (2) duration < 60 s, (3) no available GPS data (time gaps) of > 30 s between each datapoint and the preceding datapoint. GPS data were exported as latitude, longitude, and trip-characteristics. Finally, accelerometer- and GPS data were matched based on timestamp of the accelerometer. Trip mode was based on the 90th percentile speed-thresholds of 1, 10- and 35 kmph for walking, cycling and vehicle respectively [52].

### Data analyses

**Fig. 2:**
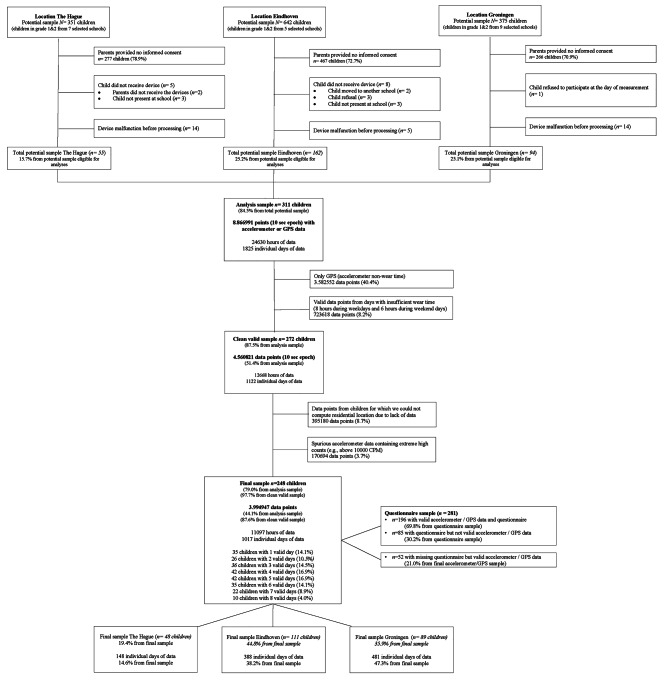
Flowchart

In total, 358 parents (26.2% from total potential sample) provided written informed consent for their child to participate in combined accelerometer and GPS measurements. After accounting for participant refusal and device malfunctioning, our sample of analysis consisted of 311 4-6-year-old children (84.5% from the sample of parents with informed consent, see Fig. 2). Next, a total of 281 parents filled in the questionnaire at the start of the study and 248 children provided valid combined accelerometer- and GPS data (i.e. sensor-data), defined as weekdays with 8 h- and weekend days with 6 h of combined accelerometer and GPS data. We defined these criteria because during weekend days, we observed less weartime due to a delayed start of weartime in the morning. From the 281 children with questionnaire-data, 85 children had insufficient sensor-data. From the 248 children with valid sensor-data, questionnaire-data were missing for 52 children. Consequently, for 196 children we had both valid sensor-data and questionnaire-data. Slight differences between the drop-out percentage between the cities were caused by the fact that in Eindhoven, accelerometers were handed out to the classroom teacher for individual children from parents that provided informed consent but were absent during the day of measurement (e.g. often due to COVID-19 restrictions). This led to an increased number of participants not meeting the 3-day valid data criteria, whereas in Groningen and The Hague, these children were considered missing a-priori and not treated as drop-out.

**Fig. 3:**
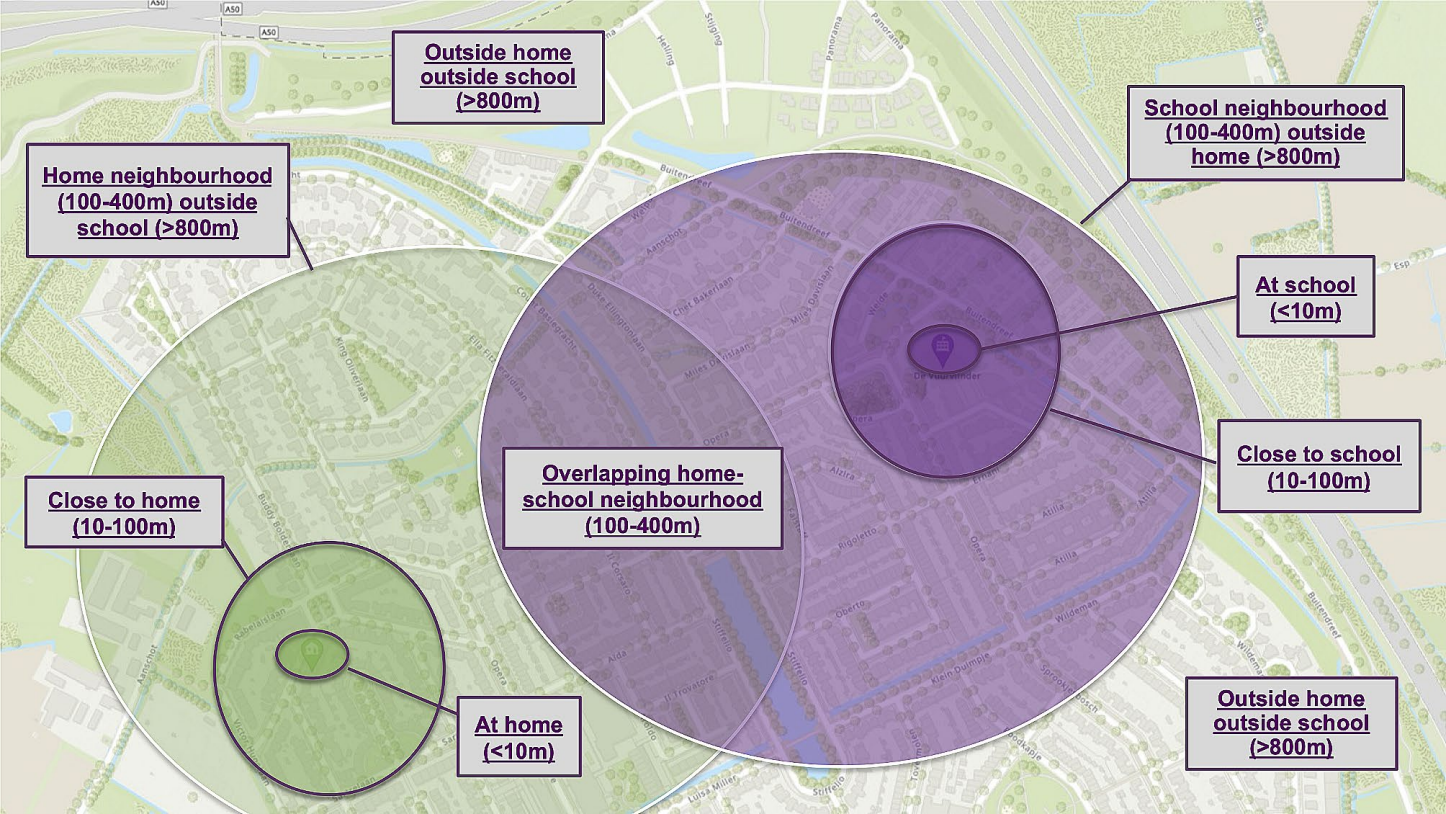
Example of distance-categories integrating both home and school locations

We imported combined accelerometer and GPS datasets for each school into ArcGIS Pro version 3.1.0 (ESRI, Redlands, CA, USA) for additional geospatial analyses. We geocoded the location of schools based on the school’s registry and extracted polygons of the school building and surrounding parcel. For the residential location of children, parents provided their six-digit postal code (i.e. identifies street-level area of 15- to 20 addresses without house number). In addition, we extracted the average centroid point of GPS data on week- and weekend days between 6- and 8 AM, during days that the child slept at home. These locations were validated by calculating Euclidean distances between the centroid point and the six-digit postal codes that parents provided in the questionnaire (median distance was 52.2 m for weekdays and 54.1 for weekend days). Next, for each datapoint, we calculated Euclidean- (i.e. < 10 m) and network distances (i.e. remaining distance categories) between children’s home and school. To investigate distances of children’s datapoints based on the combined home-school environment (not based on home and school separately), we integrated these distance-categories from both home and school (see Fig. 3). In addition, based on the Dutch national registry of large-scale topography (i.e. BGT), polygons identified as parks, sports terrains and public playgrounds were extracted and we subsequently performed ‘spatial join’ analyses to identify the datapoints that were within 10-meters from these parks, sports terrains, or playground parcels.

Parents indicated that children were awake for an average of 12 h per day and that water activities such as swimming were the primary reason for non-weartime during waking hours, while 11 parents (8.0%) reported their child experiencing discomfort when wearing the devices. Finally, only data points containing both valid accelerometer- and GPS data were retained, which resulted in a final sample of 248 children (1017 days of measurement; with 762 weekdays and 255 weekend days). We used days as the unit of analysis as this allows variation between days within children. We presented PA as minutes and percentage in light (LPA) and moderate-to-vigorous (MVPA) intensities.

## Results

Slightly more boys (54.3%) than girls participated in the study. The mean age of children was 5.56-year-old (SD = 0.75). Almost all children had either one- (57.8%) or two or more siblings (34.5%), and 61 children (40.0%) had at least one older sibling. Parents reported that 82.3% of the children slept home for all days during measurement. In total, 49.8% reported that the child had visited afterschool childcare at school for at least one day during measurement and 15.7% had visited afterschool childcare outside the school’s parcel (e.g. childcare at other location or other organization). Regarding the use of bicycles, 45.2% indicated that their child was able to cycle without supervision. In terms of organized sports, 55.6% of the children was a member of a sports club, while 30.6%- and 49.5% participated in organized sports and swimming lessons during the measurement period, respectively (see Table 1).

**Table 1:** Characteristics of the study population

	Total questionnaire sample (*N* = 281)	Combined questionnaire and sensor-data (*N* = 198)
	*N* (%)	*N* (%)
**Child demographics**		
Age at first day of measurement (mean (SD))	5.56 (0.75)	5.52 (0.74)
Gender (% boy/male)	150 (54.3%)	111 (56.1%)
**Family environment**		
Siblings (missing *n* = 6)		
No siblings	21 (7.6%)	16 (8.1%)
1 sibling	159 (57.8%)	122 (61.9%)
2 or more siblings	95 (34.5%)	59 (29.9%)
Children with older siblings (missing *n* = 2)		
No older siblings	103 (35.2%)	76 (38.8%)
1 older sibling	118 (40.3%)	90 (45.9%)
2 or more older siblings	51 (17.4%)	30 (15.3%)
**Parent-reported child behaviour**		
Slept home during all days of measurement (missing *n* = 16)	218 (82.3%)	151 (79.1%)
Child went to childcare before school hours for at least one day during measurement	31 (11.0%)	24 (12.0%)
Child went to afterschool childcare at school for at least one day during measurement	140 (49.8%)	106 (53.5%)
Child went to afterschool childcare outside school for at least one day during measurement	44 (15.7%)	30 (15.2%)
Travel to school (missing *n* = 8)		
Together with parents and/or siblings	269 (98.5%)	192 (98.0%)
Ability to ride a bicycle (missing *n* = 5)		
No, my child cannot cycle	63 (22.8%)	73 (37.2%)
Yes, only with supervision	88 (31.9%)	42 (21.4%)
Yes, without supervision	125 (45.2%)	81 (41.3%)
Member of sports club = yes (missing *n* = 6)	153 (55.6%)	114 (58.2%)
Child participated in organized sports for at least one day during measurement	86 (30.6%)	68 (34.4%)
Child participated in swimming lessons for at least one day during measurement	139 (49.5%)	94 (47.5%)
Parent allows child to play outside in the neighbourhood with supervision of siblings or peers (missing *n* = 4)	188 (67.9%)	137 (69.2%)
Parent allows child to play outside independently in the neighbourhood (missing *n* = 5)	138 (50.0%)	102 (51.5%)
Parent allows child to travel with supervision of siblings or peers to meet family or friends	117 (42.4%)	88 (44.4%)
Parent allows child to travel independently to visit family or friends	77 (27.4%)	58 (29.7%)

School start times were 8.30 am (19 schools) and 8.40 am (2 schools), while school bell times ranged from 2.00 pm to 3.15 pm. In total, 15 schools used a shortened schedule on Wednesdays (i.e. bell times ranging from 12.00 am to 12.35 pm) and 5 schools used a shortened schedule on Fridays (i.e. bell times ranging from 12.00 am to 12.30 pm). All schools provided breaks at the school parcel, so children were not allowed to leave school before school bell time. On average children lived at 2.76 km (SD = 0.33 km) pedestrian network-distance from their school (median = 604 m). Alternatively, when categorized in distance-categories, 30.8% lived within 400 m, 29.1% lived between 400- and 800 m (i.e., approximately 8 min walking time) and 40.1% lived more than 800 m from their school.

**Fig. 4:**
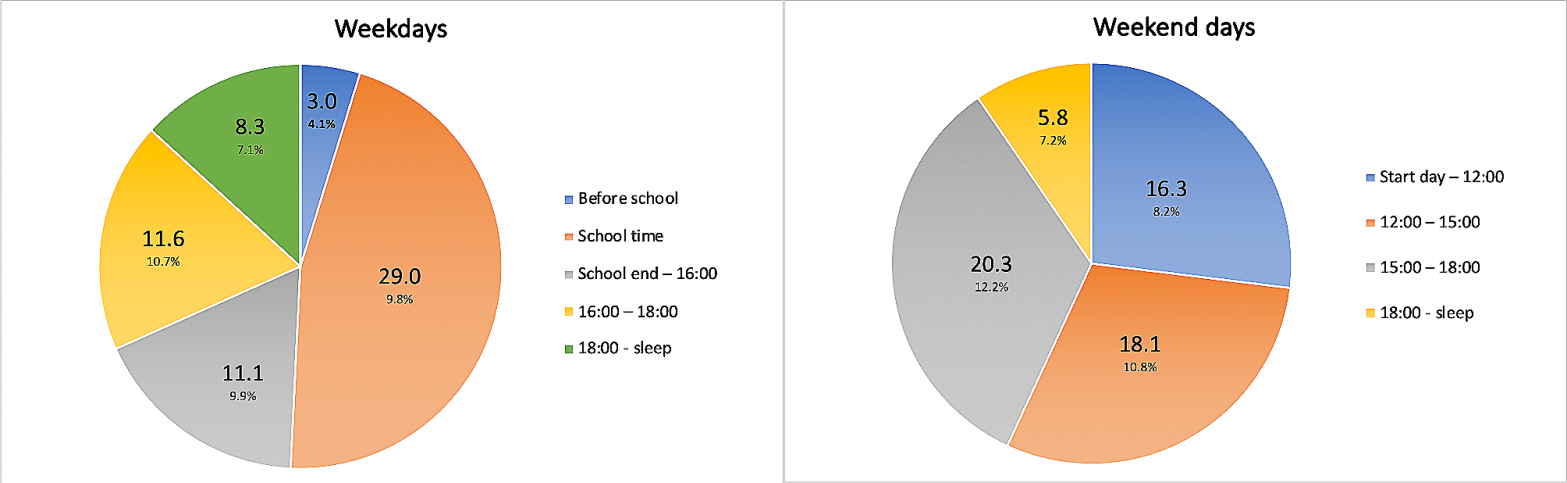
Temporal distribution of mean daily minutes of MVPA in week- and weekend days

When looking at the temporal distribution of PA, average daily weartime of combined sensor-data was 713.26 min (SD = 116.07) during weekdays and 670.34 min (SD = 117.38) during weekend days, while children performed an average of 63.00 min (8.9%) and 65.37 min (9.8%) of MVPA during week- and weekend days, respectively. On weekdays, children spent an average of 294.88 min (SD = 78.90) in the temporal school-schedule, which makes the average distribution of time during weekdays approximately 50% for combined before- and in school and 50% for afterschool till sleep (data not shown). Within weekdays, schooltime contributed to almost half of the daily MVPA (i.e. 29 min), while after school time periods approximately contributed to the other half (Fig. 4). The minutes of MVPA after school, as well as its relative percentage, gradually declined during the day. During weekends, the absolute and relative contribution of MVPA slightly increased across the day, with the most active part in the early afternoon. After 16:00 h, intensity of MVPA dropped to 7.2% on average.

When looking at the geographical distribution of PA during weekdays, percentages of LPA and MVPA were about twice as high at school versus at home (Table 2). At school, children spent on average 21.99 min in MVPA, which is 10.7% from the total daily weartime at school. Very little time was spent in the overlapping home-school neighbourhood and in the home neighbourhood outside the school neighbourhood. During weekdays, the vast majority of weartime was spent at- or close to home and school parcels. Children reported most daily minutes of MVPA at their school-parcel (i.e. approximately 22 min). Another 5.8 min of MVPA occurred within 100 m from their school, summing up to approximately 28 min. Also, compared to all other environments, the absolute- and relative contribution of LPA was highest at school, meaning that children were least sedentary at- and around their school (data not shown). At home, absolute- and relative contributions of LPA, as well as MVPA, were lower. Children spent more LPA and MVPA outside their home (but within 100 m from home) compared to their direct home location. In addition, children spent on average 70 min outside the home-school neighbourhood, with a relatively high amount of 7.5 min in MVPA (i.e. 10.6% of time spent outside home-school neighbourhood). Obviously, during weekend days the influence of school on PA disappeared, but this resulted in higher absolute- and slightly higher relative contribution of the home location in children’s LPA and MVPA (Table 2). Children spent especially more time at the ‘close to home’ location, resulting in approximately 23 min of MVPA. The percentage of MVPA that occurred at home remained relatively stable (i.e. 4.5% during weekdays versus 5.8% during weekend days). During weekends children also spent more time outside the home-school neighbourhood, while the percentage MVPA remained stable compared to weekdays. This resulted in another 23 min of MVPA performed outside the home-school neighbourhood (i.e. 12.0% of time spent at this location).

**Table 2:** Spatial distribution of mean daily minutes LPA and MVPA in weekdays and weekend days

	Weekdays			Weekend days		
	Weartime at location (SD)	LPA (SD) and % from weartime at location	MVPA (SD) and % from weartime at location	Weartime at location	LPA (SD) and % from weartime at location	MVPA (SD) and % from weartime at location
At home (< 10 m)	130.08 (88.75)	37.12 (35.16) 28.5%	5.80 (6.37) 4.5%	176.15 (110.90)	65.41 (53.01) 37.1%	10.24 (9.52) 5.8%
Close to home (10–100 m)	152.19 (101.37)	61.00 (47.75) 40.1%	11.89 (12.34) 7.8%	249.30 (155.95)	117.55 (83.48) 47.2%	23.59 (23.16) 9.5%
Home neighbourhood (100–400 m) and outside school neighbourhood (> 800 m)	1.17 (5.39)	0.69 (3.43) 59.0%	0.14 (1.28) 12.0%	1.71 (14.35)	1.18 (10.57) 69.0%	0.23 (2.59) 13.5%
At school (< 10 m)	205.19 (149.92)	129.77 (94.77) 63.2%	21.99 (22.06) 10.71%	2.04 (22.98)	1.18 (13.31) 57.8%	0.20 (1.82) 9.8%
Close to school (10–100 m)	62.08 (88.47)	36.42 (49.20) 58.7%	5.81 (9.57) 9.4%	13.83 (78.12)	6.19 (31.07) 44.8%	1.08 (5.18) 7.8%
School neighbourhood (100–400 m) and outside home neighbourhood (> 800 m)	19.18 (61.03)	10.71 (34.48) 55.8%	2.32 (8.62) 12.1%	9.70 (40.05)	4.02 (16.29) 41.4%	0.75 (4.31) 7.7%
Overlapping home-school neighbourhood (both 100–400 m)	1.29 (10.08)	0.82 (6.51) 63.6%	0.28 (2.34) 21.7%	0.44 (2.87)	0.31 (2.34) 70.5%	0.03 (0.24) 6.8%
Outside home and school neighbourhood (> 800 m)	71.39 (101.20)	25.69 (52.50) 36.0%	7.54 (15.47) 10.6%	193.30 (175.30)	97.53 (86.12) 50.5%	23.16 (30.53) 12.0%

Transport-related pedestrian trips were responsible for approximately 45% (i.e. 26 min) and 38% (i.e. 22 min) of children’s average daily MVPA during weekdays and weekend days respectively (Table 3). Higher daily mean minutes of pedestrian trips were found during weekdays compared to weekends. During weekdays, additional analyses revealed that slightly more minutes of daily pedestrian trips were observed during in-school time zones (82.25, SD = 80.22 min) compared to afterschool time zones (66.29, SD = 61.36 min). The influence of bicycle- and motorized trips to MVPA was substantially lower. In general, this also means that approximately 30 min of MVPA during weekdays- and 34 min during weekend days was spent relatively stationary (i.e. not identified by GPS-based algorithm as a transport trip). The percentage MVPA was higher in pedestrian trips compared to stationary activities (e.g. 12.3% versus 6.8%, respectively).

**Table 3:** Mean daily minutes LPA and MVPA performed in transport-trips, parks, sports terrains and playgrounds

	Weekdays			Weekend days		
	Weartime in context	LPA (SD) and % from weartime in context	MVPA (SD) and % from weartime in context	Weartime in context	LPA (SD) and % from weartime in context	MVPA (SD) and % from weartime in context
Not in trip: stationary	454.45 (140.36)	207.63 (79.53) 45.7%	30.85 (17.45) 6.8%	444.42 (120.79)	189.44 (70.31) 42.6%	34.24 (20.77) 7.7%
Transport trip: pedestrian (< 10 kmph)	214.06 (108.16)	119.96 (58.83) 56.0%	26.34 (20.42) 12.3%	154.26 (72.20)	85.28 (40.97) 55.3%	21.96 (15.87) 14.2%
Transport trip: bicycle (10–25 kmph)	16.95 (12.34)	9.53 (7.53) 56.2%	0.68 (1.01) 4.0%	19.28 (17.06)	11.03 (11.46) 57.2%	0.56 (1.00) 2.9%
Transport trip: motorized (> 25 kmph)	7.46 (14.11)	2.54 (4.43) 34.0%	0.07 (0.38) 1.0%	27.05 (44.92)	6.17 (8.85) 22.8%	0.12 (0.94) 0.4%
Public space: parks	0.61 (6.86)	0.27 (3.01) 44.3%	0.05 (0.52) 8.2%	1.11 (11.95)	0.73 (7.58) 65.8%	0.29 (3.98) 26.1%
Public space: Sports terrains	0.47 (5.30)	0.18 (2.50) 38.3%	0.02 (0.26) 4.3%	0.21 (3.67)	0.01 (0.08) 4.8%	< 0.01 (0.01) 0.05%
Public space: Playgrounds	2.79 (17.05)	1.38 (7.33) 49.5%	0.44 (3.01) 15.8%	5.09 (25.82)	3.14 (15.84) 61.7%	0.50 (2.36) 9.8%

Public open spaces equipped for PA (i.e. parks, sports terrains, and playgrounds) played a minor role in young children’s daily PA patterns. Although playgrounds showed a relatively high percentage of time spent in MVPA, absolute time spent at playgrounds was relatively low (2.8 and 5.1 daily minutes during week- and weekend days, respectively).

## Discussion

This study demonstrated context-specific PA patterns of young children by investigating their PA through space and time. More specifically, we showed that at the onset of primary school, half of children’s daily amount of MVPA during weekdays occurred at school or within 100 m from school, while the other half was divided between home or within 100 m from home and environments outside children’s home-school neighbourhood. During weekend days, from the daily amount of MVPA (i.e., approximately 65 min), slightly over half was performed at home or within 100 m from home. Only a marginal part of total daily MVPA occurred outside the home-school neighbourhood. These findings are in line with the study of Bai and colleagues, who showed that about 60% of 3-year-old children’s daily weekday MVPA of approximately 76 min occurred within 500 m from their home [41]. Furthermore, this shows that although the school-context was responsible for over 50% of MVPA during weekdays, these children seem to be able to reallocate this with PA around home and outside the home-school neighbourhood during weekend days. This is not in line with the Structured Days Hypothesis [53], stating that the presence of structure and routine of pre-scheduled activities (e.g. physical education, active travel, limited screen time) may positively influence children’s PA. Future studies are encouraged to further unravel within-person mechanisms (both between-day and within-day) in order to tailor future PA interventions [54, 55].

Our study demonstrated the importance of pedestrian-trips in daily MVPA of young children. Urban planners, school boards, policy-makers and health scientists are encouraged to co-develop initiatives that persuade parents and children to use active mobility instead of passive forms while exploiting the potential of supportive social- and physical environments [56]. Sensitivity analyses revealed that during weekdays most of time spent in pedestrian trips were during school time. However, we also showed that in our sample, cycling played a minor role in daily MVPA, which is in contrast with studies in older Dutch children [57] showing that cycling being one of the major contributors to daily PA in Dutch children [58]. This is in accordance with the long history of normalization of daily cycling mobility in the Netherlands [59, 60]. Children usually learn to cycle around the age of 5–7 years [61]. According to our questionnaire-data, parents reported that most of the children in our study was technically already able to ride a bike with- (32%) or without supervision (45%), but 98.5% reported supervision of parents- or siblings in home-school trips. An alternative explanation for this finding may be the use of the uniaxial signal of our hip-worn accelerometer in our study, as this underestimates PA during cycling [62]. Future studies, especially in older populations, are encouraged to improve measurement of cycling (e.g. using alternative placement, tri-axial signals, or multiple measurements). In addition, future studies may continue to distinguish between transport trips and relative stationary PA (i.e. not identified as a trip), potentially also leading to associations with motor development of young children.

The present study contributed to the understanding of how children’s integrated school- and home environments contribute to their daily PA, in both week- and weekend days. Previous studies have investigated PA from either one of these environments [63, 64], but to our knowledge, this is the first study that applied this combination of contexts. In particular, this study showed that especially during weekends, a considerable proportion of MVPA was performed > 800 m from both home- and school locations. This is again in line with preschool-data from Bai and colleagues, who showed that almost 30% of daily MVPA occurred at residential locations outside children’s neighbourhoods [41]. Our data showed that young children’s daily exposure during both week- and weekend days in parks, sports terrains, and playgrounds was very low but the percentage of MVPA at these locations was relatively high. This may require specific interventions focusing on increasing young children’s exposure at these environments, potentially as a multi-component involving both the home/family- and the school setting [65]. In addition, based on the same findings regarding the low daily minutes of PA that occurred in parks, sports terrains, and playgrounds, it seems unlikely that MVPA outside home-school neighbourhood would relate to these specific locations. Furthermore, it seems also unlikely that afterschool childcare or care by grandparents outside the neighbourhood may be responsible for this, since our questionnaire-data showed that only 15% of the parents reported a visit to non-school childcare for at least one day during measurement. Another suggestion may be that these children often participated in pre-arranged play sessions at a friend’s house outside their own neighbourhood or more informal play-spaces around their residential neighbourhood, but future research should provide additional insight in this type of affordance [41].

One of the strengths of this study is the inclusion of multiple study-sites surrounding three cities in the Netherlands, which allowed us to study children’s PA patterns in diverse settings, increasing the variability in environmental exposure [34]. In addition, the use of the combined accelerometer GPS methodology allowed us to objectively monitor context-specific PA patterns throughout multiple days, minimizing potential recall bias. The additional use of geospatial analyses yielded further understanding of where young children are active. Although efforts were made to include a diverse and representative sample of young children by recruiting schools from multiple Dutch cities and the fact that daily PA of our sample was relatively comparable to international literature, it still may be that wearing accelerometer- and GPS devices was most interesting for active children or parents that perceive their child as relatively active. Future technological advancements such as smaller wrist-worn devices may have potential to be suitable and interesting for all children. Another potential weakness of this study was the use of a descriptive approach that elaborated on mean daily patterns for all children in multiple contexts, while future studies may implement a more evaluative approach to investigate differences between subgroups of children or evaluate determinants of specific behaviours (e.g. active transport to- and from school) or environments. For example, the relative contribution of school times to children’s daily PA may vary between types of children and environments where they live, allowing increased tailoring of PA intervention to the target group. We showed that MVPA at- and around children’s home was low. As previous research indicated that there is a lack of knowledge about facilitators and barriers in the home-based family environment (e.g. related to practices of both active and sedentary behaviours) [66] Previous studies showed that parents act as key gatekeepers for children’s spatial freedom [67, 68], while this study demonstrated the importance of the environment within 100 m from home. Hence, it seems essential to get a better understanding of how parental rearing-constructs such as perception of traffic safety or ‘stranger danger’, but also social- and environmental factors influence parent-practices regarding independent mobility and, in turn, influences children’s PA [69]. Indications from our questionnaire data show that approximately 50% of the parents allowed their child to independently play in their neighbourhood, while 27% allowed their child to independently travel to visit family or friends. Supervision of siblings or peers increased the percentages above to approximately 68% and 42%, respectively. Future studies are encouraged to progress this field by combining data from parents (e.g. child-rearing constructs) and objective PA- and location data from children, with specific interest for home and school environments.

Our sample of 5.5-year-old children reported approximately 29 min of weekday MVPA during schooltime. Conversely, a previous review suggested that in older children, less than a quarter reached 30 min schooltime MVPA and that adolescents even reported lower levels [70]. Additionally, a previous study showed that in a sample of 7-11-year-old children with relatively low motor competence, school was the least active time period of their day. compared to before- and after school [71]. In turn, recent research showed that longer-term integration of PA in curricula, such as active breaks and physically active learning, fosters important pre-requisites of academic learning (e.g. time on task) [72, 73]. Therefore, schools are well-suited for addressing important PA-related health inequalities of young children [74] and are therefore encouraged to implement evidence-based policies and to systematically evaluate which parts of the school-system hamper and stimulate their pupil’s PA as well as academic performance.

## Conclusions

Overall, our study demonstrated the importance of schools in supporting PA of young children during weekdays. During weekends, the environment within 100 m from young children’s home was important, as well as locations outside the home-school neighbourhood. During- week and weekend days, walking contributed to almost half of the daily MVPA, emphasizing the importance of active school transportation- but also habitual daily walking and cycling (during week- and weekend days) for sustainable and PA promotion in children.

## Data Availability

No datasets were generated or analysed during the current study.

## Abbreviations

NWO: Dutch Organization for Scientific Research
PA: Physical Activity
MVPA: Moderate-to-vigorous Physical Activity
GPS: Global Positioning System
GIS: Geographic Information System
PALMS: Personal Activity Location Measurement System
HABITUS: Human Activity Behavior Identification Tool and data Unification System
GGIR: Software R-package to process raw accelerometer data
SD: Standard Deviation
